# Flavone-rich maize: an opportunity to improve the nutritional value of an important commodity crop

**DOI:** 10.3389/fpls.2014.00440

**Published:** 2014-09-08

**Authors:** María I. Casas, Silvia Duarte, Andrea I. Doseff, Erich Grotewold

**Affiliations:** ^1^Center for Applied Plant Sciences, The Ohio State UniversityColumbus, OH, USA; ^2^Molecular Cellular and Developmental Biology Graduate Program, The Ohio State UniversityColumbus, OH, USA; ^3^Department of Molecular Genetics, The Ohio State UniversityColumbus, OH, USA; ^4^Division of Pulmonary, Allergy, Critical Care, and Sleep, Department of Internal Medicine, Heart and Lung Research Institute, The Ohio State UniversityColumbus, OH, USA

**Keywords:** maize, metabolism, nutrition, inflammation, cancer, nutritional disparity

## Abstract

Agricultural outputs have resulted in food production continuously expanding. To satisfy the needs of a fast growing human population, higher yields, more efficient food processing, and food esthetic value, new crop varieties with higher caloric intake have and continue to be developed, but which lack many phytochemicals important for plant protection and adequate human nutrition. The increasing incidence of chronic diseases such as obesity, diabetes and cardiovascular diseases, combined with social disparity worldwide prompted the interest in developing enhanced crops that can simultaneously address the two sides of the current malnutrition sword, increasing yield while providing added nutritional value. Flavones, phytochemicals associated with the beneficial effects of the Mediterranean diet, have potent anti-inflammatory and anti-carcinogenic activities. However, many Mediterranean diet-associated vegetables are inaccessible, or lowly consumed, in many parts of the world. Maize is the most widely grown cereal crop, yet most lines used for hybrid maize production lack flavones. As a first step toward a sustainable strategy to increasing the nutritional value of maize-based diets, we investigated the accumulation and chemical properties of flavones in maize seeds of defined genotypes. We show that the pericarps of the *P1-rr* genotype accumulate flavones at levels comparable to those present in some flavone-rich vegetables, and are mostly present in their *C*- and *O*-glycosylated forms. Some of these glycosides can be readily converted into the corresponding more active health beneficial aglycones during food processing. Our results provide evidence that nutritionally beneficial flavones could be re-introduced into elite lines to increase the dietary benefits of maize.

## Introduction

Malnutrition is widely spread worldwide, presented in diverse forms from undernourishment in people without access to minimum diet requirements, to people with access to processed foods rich in fats and sugars, dramatically increasing diet-related chronic diseases such as obesity, diabetes and cardiovascular diseases (Tanumihardjo et al., 2007; Martin et al., 2013). Hence, there is a growing interest to improve food nutritional value in order to fulfill minimum requirements, as well as to prevent diet-associated diseases.

The Mediterranean's diet consists of a largely plant-based dietary pattern associated with the low incidence of coronary heart disease, certain cancers and other diet-related illnesses, including obesity and diabetes (Stafford, 1990; Gates et al., 2009). Its composition includes legumes, nuts and olive oil, making it markedly different from the Western-diet rich in saturated fatty acids derived from animal food (Trichopoulou and Vasilopoulou, 2000; Wahrburg et al., 2002). However, many components of the Mediterranean's diet are inaccessible or have a high cost, making other plants and plant-derived foods containing similar nutritional components potential targets to bring about improved nutritional value (Bonaccio et al., 2011).

Maize is one of the most important cereal crops worldwide, with the USA being the top producer. In order to satisfy the demands of a rapidly increasing population, most of the maize grown for feedstock, bio-ethanol and other industrial purposes are high-yield varieties grown at high densities. Additionally, to increase production, more efficient food processing methods have been developed (Morris and Sands, 2006). In order to fulfill these requirements, breeding has generated maize varieties that have looser husks, bigger endosperms, thinner pericarps and which have lost many of the phytochemicals important for plant protection, human and livestock nutrition (Bailey and Bailey, 1938; Huang et al., 2002).

Amongst the phytochemicals lost during maize domestication and breeding are the flavonoids, plant specialized metabolites that play many important roles in plant growth and development, including pollen development and viability, UV protection, and insect pest deterrence (Grotewold, 2006; Falcone Ferreyra et al., 2012). Flavones, a flavonoid sub-family, have been associated with the beneficial effects of the Mediterranean diet. Specifically, the flavone apigenin, abundantly present in celery, parsley and other vegetables of the Mediterranean's diet (Justesen et al., 1998; Trichopoulou and Vasilopoulou, 2000), exhibits potent anti-angiogenic, anti-inflammatory and anti-carcinogenic activities (Vargo et al., 2006; Gonzalez-Mejia et al., 2010; Shukla and Gupta, 2010). Moreover, apigenin restores normal metabolic function during inflammatory conditions, through the regulation of mitochondrial function (Duarte et al., 2013). The identification of human cellular targets of apigenin provided new insights into the molecular mechanisms by which this flavone exerts its beneficial effects. Apigenin binds to the heterogeneous nuclear ribonucleoprotein A2 (hnRNPA2) and affects its multimerization, thereby inducing changes in splicing activity (Arango et al., 2013). Flavonoids have also been identified as potentially beneficial for feedstock nutrition, for example by reducing ruminal methanogenesis, by altering meat and milk fatty acid profiles, and by reducing lipid peroxidation in meats (Patra and Saxena, 2010; Vasta and Luciano, 2011).

**Figure 1:**
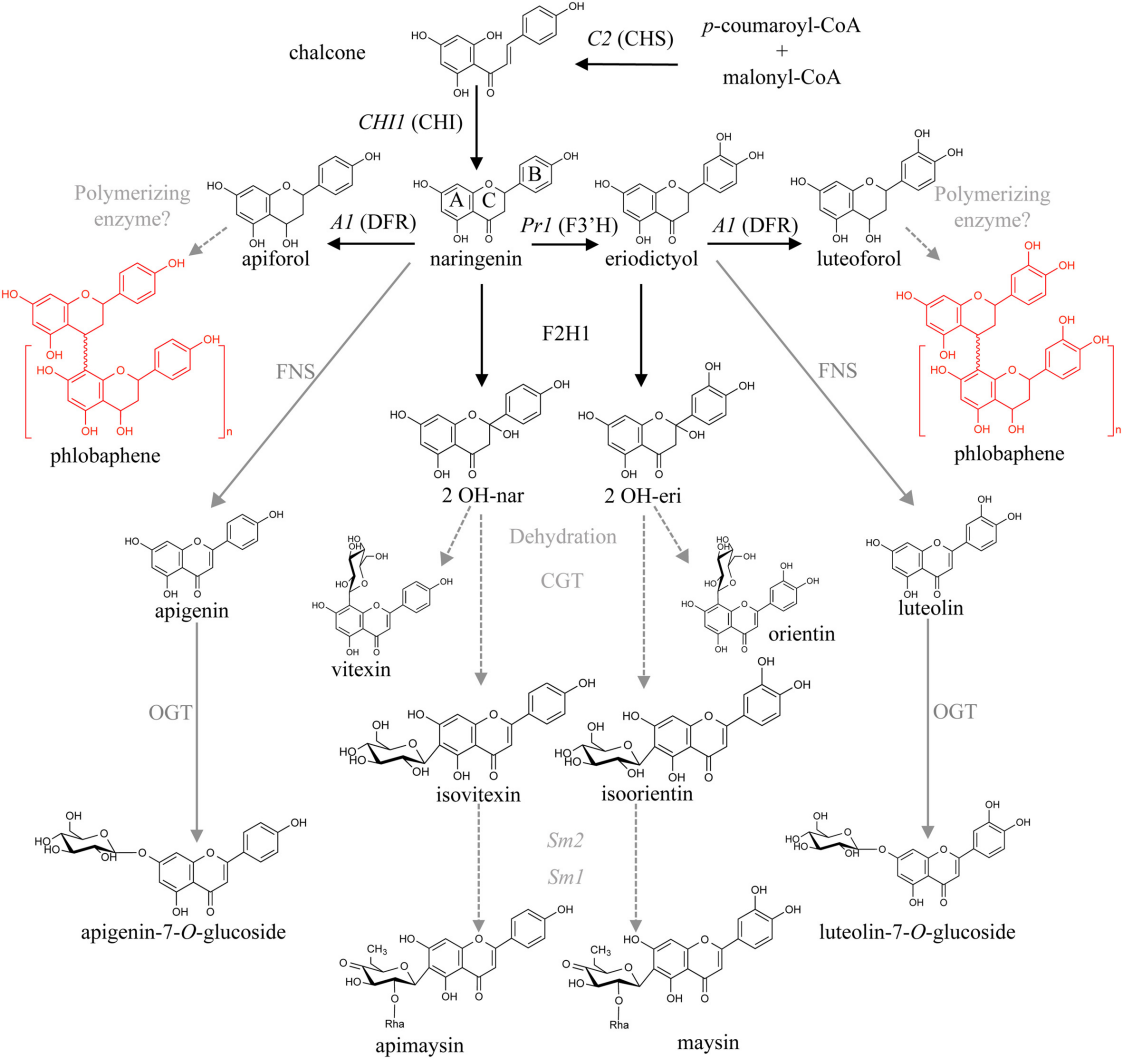
**Proposed maize flavone and 3-deoxy flavonoid biosynthetic pathways**. Condensation of *p*-coumaroyl-CoA and malonyl-CoA by chalcone synthase (referred to as CHS, encoded by the locus *C2*) results in naringenin chalcone, which is then converted to naringenin by chalcone isomerase (CHI). Naringenin is converted to apiforol (flavan-4-ol) by a dihydroflavonol reductase (DFR, *A1*) and subsequently polymerized into phlobaphenes. It can also be converted to isovitexin (*C*-glycosylflavone) by a flavanone-2-hydrohylase (F2H) and a *C*-glycosyl transferase (CGT). A flavone synthase (FNS) could also catalyze this step followed by an *O*-glycosyl transferase (OGT). Naringenin can also be converted to eriodictyol by a flavanone-3′-hydroxylase (F3′H, *Pr1*). The proposed steps for conversion of apigenin and luteolin into the *C*-glycosylflavones apimaysin and maysin respectively, involve at least three enzymatic conversions: glycosylation at C-6, followed by a rhamnosylation and dehydration steps mediated by the *Sm2* and *Sm1* loci (adapted from Morohashi et al., 2012). Enzymes identified in black, those proposed in gray.

Flavonoid biosynthesis begins with the condensation of three malonyl-CoA molecules with *p*-coumaroyl-CoA by chalcone synthase [abbreviated CHS, encoded by the *colorless2* (*C2*) locus], to form naringenin chalcone. Subsequently, chalcone isomerase (CHI) converts narigenin chalcone into the flavanone naringenin, generating a key branching step in the pathway (Winkel-Shirley, 2001; Grotewold, 2006). Naringenin can serve as substrate for the flavanone-3′-hydroxylase enzyme, encoded by the locus *Pr1*, to generate eriodictyol. Naringenin is then converted into various flavones by a combination of steps involving a flavanone-2-hydroxylase (F2H) and a *C*-glycosyl transferase (CGT), to generate apigenin-6-*C*-glucoside (or its isomer, apigenin-8-*C*-glucoside), also known as isovitexin and vitexin, respectively. In the case of eriodictyol, the flavones generated after these conversions steps are luteolin-6-*C*-glucoside (or its isomer, luteolin-8-*C*-glucoside), namely isoorientin and orientin, respectively (Winkel-Shirley, 2001; McMullen et al., 2004; Morohashi et al., 2012; Falcone Ferreyra et al., 2013). Flavones can also be generated from naringenin by means of a 2-oxoglutarate dependent dioxygenase (2-ODD), as is the case for Apiaceae like celery (Yan et al., 2014). Flavones are often *O*- or *C*- glycosylated by glycosyl transferases, to generate, for instance, apigenin-7-*O*-glucoside (A7OG, Figure 1).

**Figure 2:**
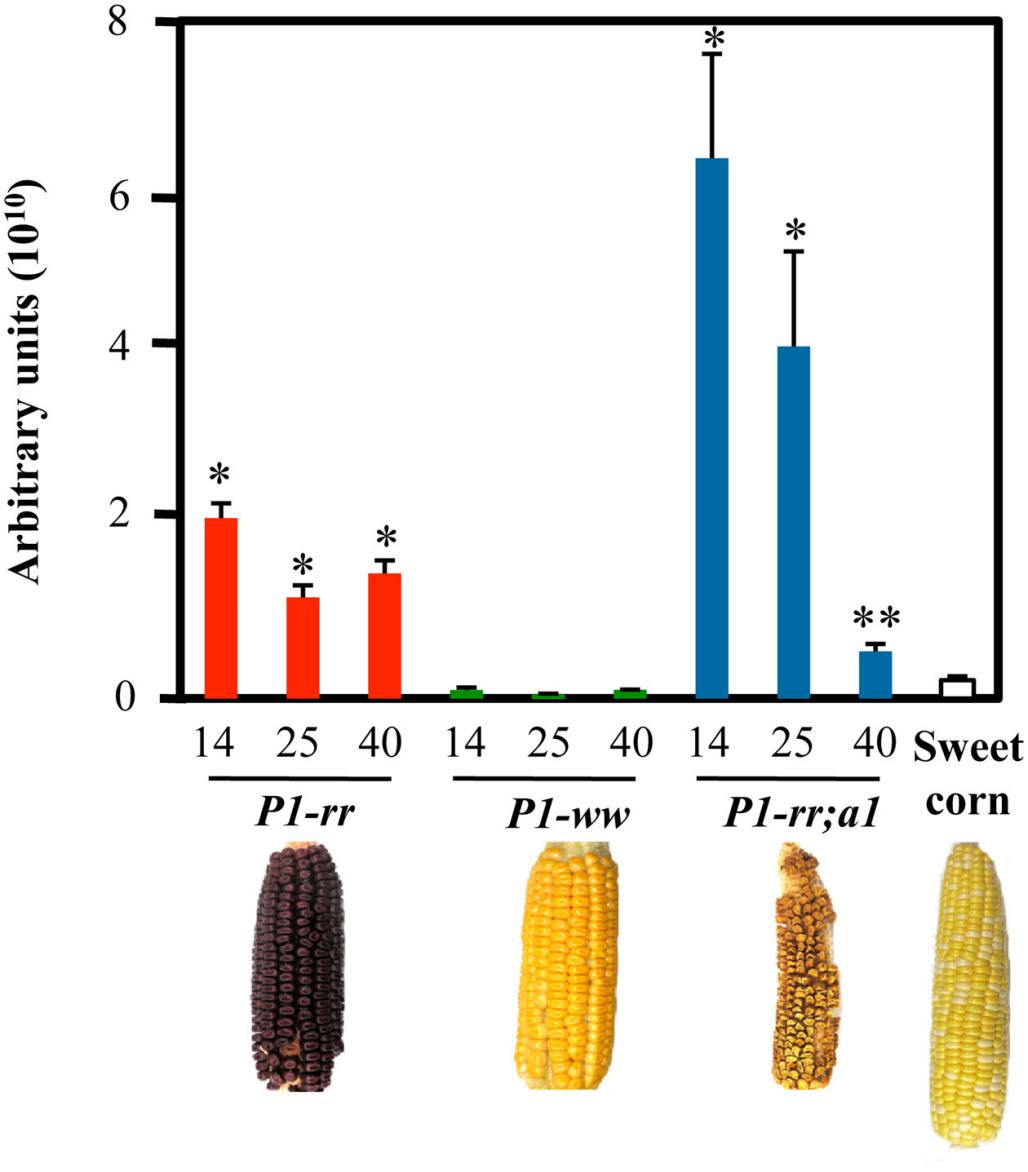
**Higher flavonoid levels in *P1* expressing lines**. HPLC analyses of pericarps at 14, 25, and 40 DAP. Total flavonoid levels are indicated as arbitrary units (mean values ± s.e.m., *n* = 5 for all maize lines and *n* = 3 for *P1-rr;a1*). *t*-test ^*^p < 0.01 or ^**^p < 0.05 compared to sweet corn. Arbitrary units correspond to total peak area at 350 nm per g of dry pericarp.

The R2R3-MYB transcription factor PERICARP COLOR 1 (P1) controls the accumulation of the brick red phlobaphene pigments in the pericarp (Styles and Ceska, 1977; Grotewold et al., 1991b) the outermost layer of the kernel, a maternal tissue that corresponds to the modified ovary wall (Kiesselbach, 1949). Phlobaphenes are polymers of the flavan-4-ols apiforol or luteoforol, generated from naringenin or eriodictyol by dihydroflavonol reductase (DFR), encoded by maize *A1*. Different *P1* alleles specify different pericarp and cob glume colors. For example, *P1-ww* results in white pericarps and white cob glumes, whereas *P1-rr* specifies red pericarps and red cob glumes (Figure 2). *P1-wr* and *P1-rw* alleles responsible for white pericarps and red cobs, or red pericarps and white cobs respectively, have also been described (Anderson, 1924; Chopra et al., 1996). *A1* mutants in a *P1-rr* background (*P1-rr;a1*) display an unidentified brown pigment that contrasts with the white *P1-ww* pericarps, suggesting metabolic shunting toward a different branch of the flavonoid pathway (Styles and Ceska, 1977). Additionally, *P1* has been identified as a major QTL for *C*-glycosylflavone accumulation in maize silks; in the absence of a functional *A1* allele, *P1-rr* pericarps accumulate *C*-glycosylflavones (Styles and Ceska, 1977; Byrne et al., 1996). Most commercial high-yield hybrid maize lines are colorless in the pericarp and lack flavones. As is the case for sweet corn and other commercial varieties, maize kernels contain over 25% sugar (Singh et al., 2014), resulting in maize being partially responsible for the poor nutritional value combined with high caloric intake of many processed foods. For example, high fructose corn syrup has been associated with high incidence of metabolic syndrome, diabetes, kidney damage and liver disease (Ferder et al., 2010).

As a first step toward a rational strategy to increase the nutritional value of maize-based diets, we investigated the accumulation and chemical properties of flavones in defined maize genotypes. We show here that the pericarps of *P1-rr* maize accumulate flavones at levels comparable to those present in flavone-rich vegetables. We found that a significant portion of these flavones is present as *C*- and *O*-glycosylated derivatives. We describe strategies that permit the conversion of some of these glycosides into the more bioactive aglycones in a whole food context. The levels of flavones in various maize lines, including *P1-rr;a1*, were also quantified. Together, our results provide evidence that nutritionally beneficial flavones could be reintroduced into maize elite lines to increase the dietary benefits of maize products.

## Materials and methods

### Maize stocks and plant materials

The *P1-rr* 65-CFS-305 and *P1-ww* 4Co43 lines were introgressed into A619 (*P1-ww*) as previously described (Morohashi et al., 2012). The *P1-rr;a1* stock has the *P1*^4*B*−2^ allele into which the *a1::rd-t sh2* mutant allele was introduced by crossing (Grotewold et al., 1991a; Pooma et al., 2002). It is not known however, whether the *A1* allele in the *P1-rr* 65-CFS-305 line is the same as in *P1-rr;a1*. It was also noted that the kernel and ear sizes from *P1-rr;a1* were smaller, compared to those of the other lines in this study. In all cases, pericarps and whole kernels were collected at different developmental times including 14, 25, and 40 days after pollination (DAP) and saved at −80°C for further analyses. Sweet corn ears were obtained from various commercial providers at milky stage (approximately 18 DAP).

### Chemicals

Apigenin, A7OG, luteolin, L7OG, vitexin (apigenin-8-*C*-glucoside), isovitexin (apigenin-6-*C*-glucoside), luteolin and orientin (luteolin-8-*C*-glucoside) standards were obtained from Sigma (Sigma Aldrich, St. Louis, MO); isoorientin (luteolin-6-*C*-glucoside) was kindly provided by Dr. Michael McMullen (USDA-ARS, Columbia, MI). Methanol and acetonitrile were obtained from Sigma (Sigma Aldrich) and ethanol was obtained from Decon Labs, Inc. (King of Prussia, PA). HCl, H_3_PO_4_, KOH were obtained from Fisher Scientific (Fair Lawn, NJ).

### Flavonoid extraction from maize pericarps

Fresh pericarps were dissected at 14, 25, and 40 DAP and freeze-dried overnight. Dried pericarps were extracted with 100% methanol over-night at −20°C using 50 mg of dry pericarps per ml of methanol. Following incubation, half the volume of the methanolic extracts were used for further analyses (non-hydrolyzed), and the other half was dried under vacuum and re-suspended in 5% (v/v) HCl in butanol and boiled for 1 h (hydrolyzed) prior to analyses.

### Maize whole-food extract preparation

Maize extracts were prepared as similarly described (Hostetler et al., 2012). Briefly, kernels stored at −80°C were macerated with mortar and pestle and incubated for 3 h at room temperature, subsequently frozen in liquid N_2_ and lyophilized overnight. Kernels were incubated for 3 h following the same protocol as previously used for celery (Hostetler et al., 2012), which was used as a positive control for extract preparation. The lyophilized material was ground, re-suspended in 1.5 N H_3_PO_4_ (at 1 gr per 17 ml) and boiled for 90 min, before neutralizing with 10% KOH. This extract is referred hereafter as AME (acid whole maize extract). In addition, the mix was incubated with commercially available ground raw almond (Whole Foods Market, Columbus, OH; 17% dry weight of almond over 100 g of the lyophilized material) for 2 h at 50°C to convert the *O*-glucosides to aglycones. The resulting mixture was then lyophilized, and 1 gr was extracted twice with 5 ml ethanol 70% (v/v). This extract was referred hereafter as EME (enzymatically treated whole maize extract). An aliquot of both AME and EME extracts were dried under N_2_ gas and reconstituted in 100% methanol for LC-MS/MS analyses.

### HPLC analyses

Pericarps and maize whole-food methanolic extracts were filtered through a 0.2 μm membrane (Pall Nanosep® MF) prior injecting 20 μl into a W2690-5 Waters (Milford, MA) separation module and a W2996 photodiode array (PDA) module following absorbance at 350 nm, corresponding to the maximum absorbance wavelength for flavones (Snook et al., 1989) and flavonols (Anouar et al., 2012), using a Symmetry C18 reverse-phase column (3.5 μm, 4.9 mm, 75 mm, Waters). Running conditions were conducted as previously described (Casati and Walbot, 2005), briefly, an initial gradient of 80% solvent A—20% solvent B (solvent A: water; solvent B: acetonitrile) with a 0.75 ml/min flow to 100% solvent B at 15:33 min and kept for 1:20 min to finally return to initial conditions at 18:00 min. Total flavonoid levels were expressed as total peak area at 350 nm per g dry weight of pericarp tissue, referred to as arbitrary units in Figure 2. A fresh kernel weights ~150 mg at 14 DAP and the pericarp corresponds to ~20% of the total fresh weight at this developmental stage. All statistical analyses were done using two-tailed Student's *t*-test.

### Flavone LC-MS/MS analyses

Hydrolyzed and non-hydrolyzed pericarp methanolic extracts and maize whole-food methanolic extracts were used for flavone analyses performed by liquid chromatography followed by tandem mass spectrometry (LC-MS/MS) on a Linear Ion Trap Quadrupole LC-MS/MS with a Q-Trap 5500 Mass Spectrometer (AB-SciEX) at the Center for Applied Plant Sciences (CAPS) Targeted Metabolomics Laboratory (TML, http://metabolomics.osu.edu/). LC was performed on a Symmetry C18 column (3.5 μm, 4.9 mm, 75 mm, Waters) injecting 15 μl with a flow of 1 ml/min, solvent A: water, solvent B: acetonitrile following previously established protocols (Falcone Ferreyra et al., 2013). Briefly, an initial gradient of 20% solvent A—80% solvent B (solvent A: water; solvent B: acetonitrile) from 0.00 to 2.00 min; followed by 7.00 min of 20 to 60% A; then 9.10 to 11.00 min to 90% A; 0.1 min decrease from 90 to 20% A and finally 11.10 to 14.00 min 20% A was used. MS spectra were acquired using electrospray ionization (ESI) and Multiple Reaction Monitoring on the negative mode. Conditions and retention times for each compound are summarized in Supplementary Table 1. Flavone quantifications were performed based on the corresponding standard curves (Supplementary Figure 1). Limits of detection, limits of quantification, correlation coefficients (R^2^), and formula weights (in atomic mass units, amu) for each flavone are summarized in Supplementary Table 2.

## Results

### Kernels of P1 expressing maize lines have high flavonoid content

Flavonoid content was evaluated in pericarps of different maize lines including *P1-rr, P1-ww, P1-rr;a1* and commercial sweet corn, at three different developmental stages: 14 days after pollination (DAP), a developmental point close to the milky stage, when corn is normally harvested for human consumption, 25 and 40 DAP, the latter time corresponding to a stage used for long-term seed storage or livestock feed. We found that the majority of the flavonoids analyzed had very short retention times ranging from 1.8 to 4.8 min by HPLC (Supplementary Figure 2) and most likely correspond to glycosides (Bligh et al., 2013). *P1-rr;a1* showed the highest amount of total flavonoids, as compared with *P1-rr, P1-ww* and sweet corn (Figure 2), in agreement with previous studies (Styles and Ceska, 1989). The levels of flavonoids in *P1-rr;a1* were 3 fold higher than *P1-rr* at 14 DAP (6.4 × 10^10^ AU compared to 2.13 × 10^10^ respectively), *P1-rr;a1* levels were 64 fold higher than *P1-ww* (1.0 × 10^9^ AU) and 29 fold higher than sweet corn (2.2 × 10^9^ AU) at the same developmental stage. At 25 DAP, *P1-rr;a1* levels were 3 fold higher than *P1-rr* (4.2 × 10^10^ vs. 1.2 × 10^10^ AU) and 92 fold higher than *P1-ww* (4.5 × 10^8^ AU). Finally, at 40 DAP, *P1-rr;a1* levels dropped to 5.6 × 10^9^ AU whereas the levels of *P1-rr* were 1.5 × 10^10^ AU and *P1-ww* 1.0 × 10^9^ AU, respectively (Figure 2). These results show that the *P1-rr;a1* genotype has a significantly higher flavonoid content at early harvest times, providing a window of opportunity for the development of flavonoid-rich maize whole foods.

### Flavones are present primarily as *C*- and *O*-glucosides in maize kernels

**Figure 3:**
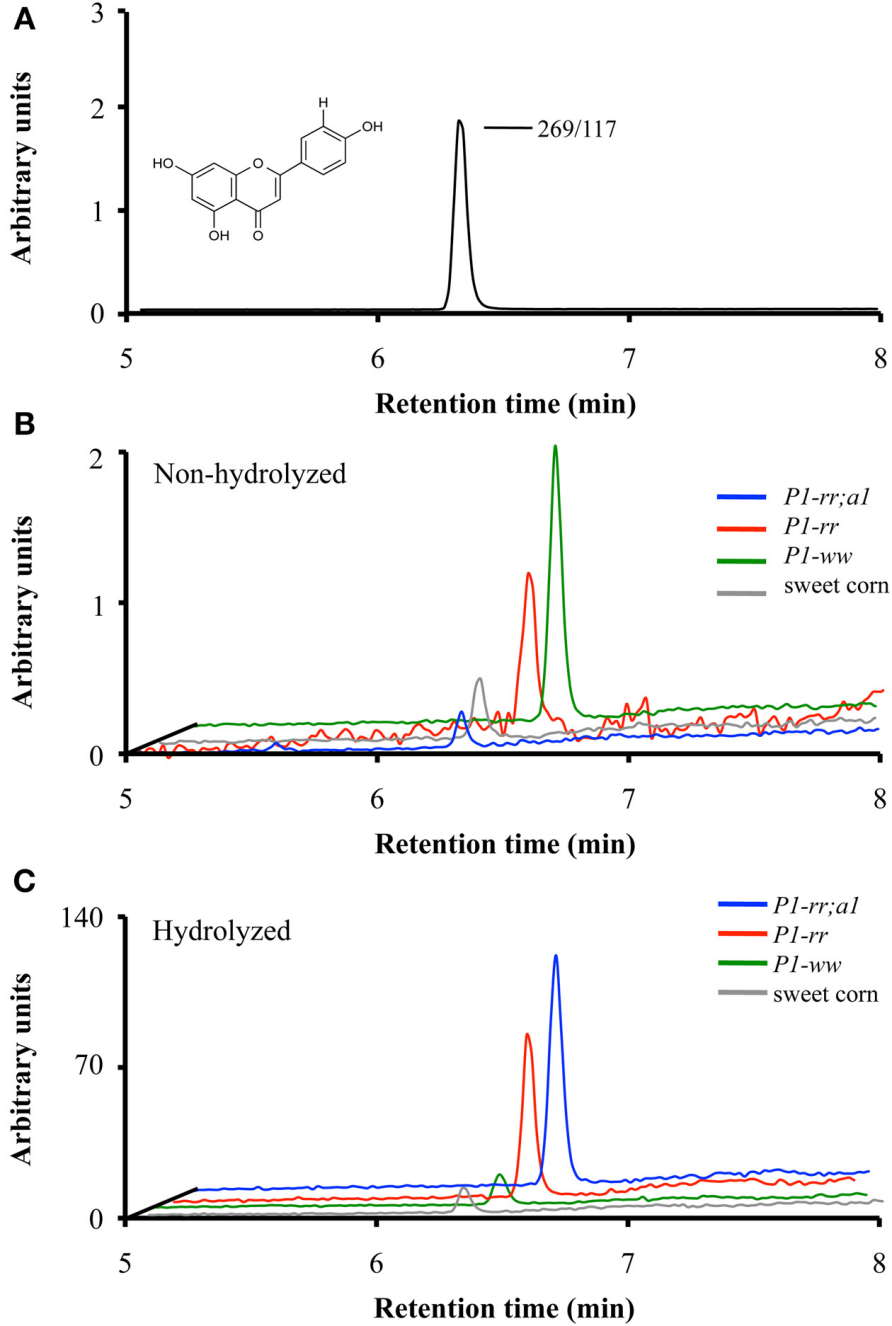
**Apigenin content in whole maize kernels**. LC-MS/MS analyses of parental/daughter transitions corresponding to apigenin (268.9/117.0) for *P1-rr* (red); *P1-rr;a1* (blue); *P1-ww* (green) at 14 DAP and sweet corn (gray) at milky stage. **(A)** Chromatogram corresponding to apigenin standard. **(B)** Chromatograms corresponding to apigenin content in non-hydrolyzed *P1-rr*; *P1-rr;a1*; *P1-ww* and sweet corn. **(C)** Chromatograms corresponding to apigenin content in hydrolyzed samples. Arbitrary units correspond to peak intensity for the 268.9/117.0 transitions in counts per second.

To identify the contribution of flavones to the absorbance observed at 350 nm in the various maize genotypes, liquid chromatography followed by tandem mass spectrometry (LC-MS/MS) was performed on pericarp extracts of the various maize lines at 14 and 25 DAP and in sweet corn at milky stage (shown in Figure 3 for apigenin). *P1-ww* and sweet corn kernels showed the lowest levels of total flavones, ranging between ~0.6 −1.3 μg flavones per 100 gr fresh kernel respectively (Table 1), consistent with the results obtained by HPLC (Figure 2). *P1-rr* accumulated 360 and 71 μg of total flavone per 100 gr of fresh kernel at 14 and 25 DAP, respectively. Notably, the introduction of the *a1* allele into *P1-rr* significantly increased (~100 fold) the levels of total flavone, resulting in 36 and 25 mg of total flavone per 100 gr of fresh kernel at 14 and 25 DAP, respectively (Table 1). We found that in all the lines, flavone content decreased during development. This is likely a consequence of the diversion of naringenin toward flavan-4-ol formation, which will polymerize into phlobaphenes in *P1-rr*, or into the brown pigment in *P1-rr;a1*, as pericarp development continues.

**Figure 4:**
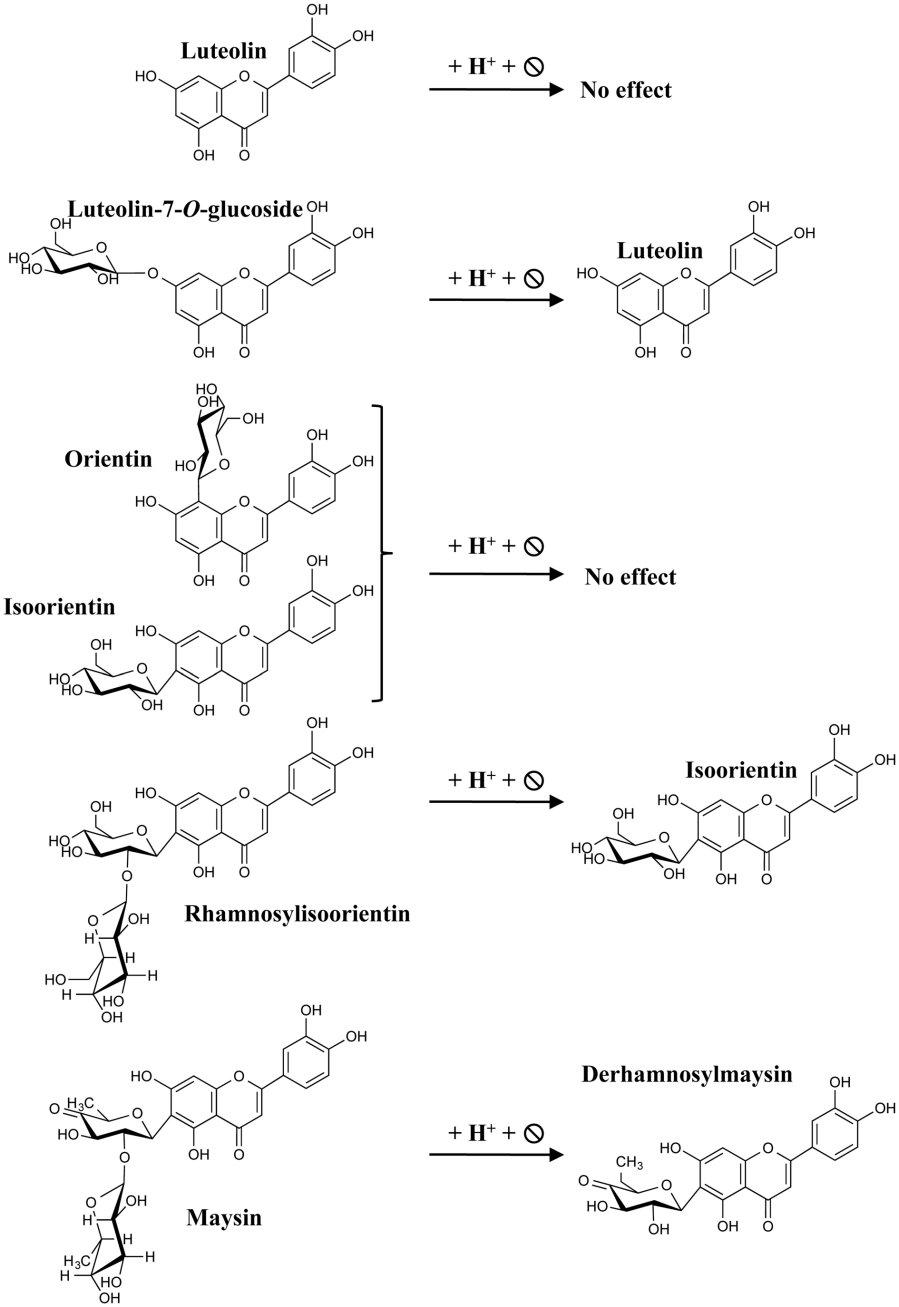
**Expected flavones resulting from acid hydrolysis**. The scheme depicts the different flavones used in this study and their respective hydrolyzed derivatives expected for luteolin.

**Table 1:** **Flavone content in non-hydrolyzed whole maize kernel**.

	***P1-ww*^a^**		***P1-rr*^b^**		***P1-rr;a1*^c^**		**Sweet corn^d^ milky stage**
	**14 DAP**	**25 DAP**	**14 DAP**	**25 DAP**	**14 DAP**	**25 DAP**	
Apigenin	0.4 ± 0.2	0.03 ± 0.01^*^	0.3 ± 0.1	0.10 ± 0.03	2 ± 2	0.5 ± 0.2	0.20 ± 0.04
Luteolin	0.03 ± 0.01	0.010 ± 0.003	0.20 ± 0.06	0.07 ± 0.02	18 ± 5	8 ± 4	0.05 ± 0.03
A7OG	0.02 ± 0.01	<0.005	13 ± 5	1.0 ± 0.3	2.4 ± 1.1	5 ± 2	0.030 ± 0.002
L7OG	0.03 ± 0.02	0.020 ± 0.003^*^	3.0 ± 0.6^*^	0.7 ± 0.1	1023 ± 342	453 ± 189	0.06 ± 0.01
IV	<0.005	<0.005	8 ± 2^*^	1.4 ± 0.3	9 ± 3	11 ± 4	<0.005
Vitexin	<0.005	<0.005	9 ± 2^*^	1.5 ± 0.3	7 ± 2	11 ± 4	<0.005
IO	0.05 ± 0.02	0.010 ± 0.003	10 ± 3	2.2 ± 0.5	1552 ± 653	1369 ± 648	0.6 ± 0.5
Orientin	<0.005	<0.005	9 ± 2	1.6 ± 0.3	116 ± 41	85 ± 32	0.07 ± 0.05
RIO	0.010 ± 0.004^*^	0.010 ± 0.003^*^	62 ± 20	16 ± 6	6779 ± 3265	7908 ± 4408	0.09 ± 0.02
Maysin	0.02 ± 0.01	<0.005	246 ± 51^*^	46 ± 4	26,291 ± 12,340	14,694 ± 7132	0.23 ± 0.16
Total	0.6 ± 0.3	0.08 ± 0.02	360 ± 85	71 ± 12	36,000 ± 17,000	25,000 ± 12,000	1.3 ± 0.9

Flavone content was determined by LC-MS/MS using MRM in the negative mode following parental/daughter transitions corresponding to flavones in their aglycone and glycosylated forms. Results are indicated as μg of flavones/100 grams of fresh kernels. t-test values

* p < 0.01 compared to sweet corn. Mean values ± s.e.m.; biological replicates for a: n = 6; b: n = 5; c and d: n = 3. A7OG, apigenin-7-O-glucoside; L7OG, luteolin-7-O-glucoside; IV, isovitexin; IO, isoorientin and RIO, rhamnosylisoorientin.

*P1-rr;a1* had the highest contents of apigenin and luteolin at both developmental stages (2 and 18 μg/100 gr fresh weight at 14 DAP, and 0.54 and 8 μg/100 gr fresh weight at 25 DAP, respectively), when compared to the other maize lines. Unexpectedly, we found that apigenin and luteolin were similar in *P1-rr* and *P1-ww* or sweet corn at both developmental stages (Table 1). The difference between apigenin and luteolin levels at both developmental stages in *P1-rr;a1* could be explained by the presence of the *Pr1* locus, encoding a flavanone-3′ hydroxylase (Sharma et al., 2011), responsible for adding a hydroxy group to C3 on the B-ring of the flavonoid backbone (Figure 1).

Apigenin-7-*O*-glucoside (A7OG) and luteolin-7-*O*-glucoside (L7OG) contents were similarly low in *P1-ww* and sweet corn (Table 1). A7OG levels at 14 DAP were 5 fold higher in *P1-rr* as compared with *P1-rr;a1*, accumulating ~13 and 2 μg/100 gr fresh weight, respectively. At 25 DAP, this difference is smaller, with *P1-rr;a1* accumulating more A7OG than *P1-rr*. L7OG was remarkably (~340 fold) higher in *P1-rr;a1* compared to *P1-rr* at 14 DAP (1023 vs. 3 μg/100 gr fresh weight), and ~ 450 fold higher at 25 DAP (Table 1).

The apigenin *C*-glucosides vitexin and isovitexin were barely detectable in sweet corn and *P1-ww* at both developmental stages (Table 1). They accumulate at similar levels in *P1-rr* and *P1-rr;a1* at 14 DAP (8 and 9 μg/100 gr fresh weight, and 9 and 7 μg/100 gr fresh weight, respectively) and no significant changes were observed in these compounds at 25 DAP in *P1-rr;a1*, whereas approximately a 6 fold decrease is observed for *P1-rr* (Table 1). The luteolin-derived *C*-glucosides, orientin and isoorientin, are found more abundantly in *P1-rr;a1* at both developmental stages, with isoorientin reaching almost an order of magnitude higher than orientin (~1500 μg vs. ~120 μg/100 gr fresh weight, respectively). In both cases, no differences are observed at 14 and 25 DAP. Orientin and isoorientin are present in *P1-rr* at reasonably high levels, but correspond to 1 or 10% respectively to the levels present in *P1-rr;a1* (Table 1).

Rhamnosylisoorientin (RIO), the precursor for the strong insect deterrent compound maysin (Byrne et al., 1998), contains the luteolin backbone (Figure 1). Our results indicate that *P1-ww* and sweet corn have low but similar levels of RIO and maysin (~0.01–0.09 μg/100 gr fresh weight). *P1-rr* has significantly higher levels of RIO and maysin, reaching ~62 and 246 μg/100 gr fresh weight at 14 DAP, and these levels decrease by more than 50% by 25 DAP (16 and 46 μg/100 gr fresh weight, respectively). In *P1-rr;a1*, a remarkably higher content of both RIO and maysin is observed, reaching ~7 and 8 mg/100 gr fresh weight for RIO at both developmental stages and as high as ~26 mg/100 gr fresh weight at 14 DAP for maysin, that decreased to almost half at 25 DAP (Table 1). Taken together, these findings show that the levels of flavones can be significantly improved by the presence of the P1 transcription factor in *P1-rr* maize lines, and that this effect is even more pronounced in lines that have the recessive *a1* allele.

### Acid hydrolysis increases maize kernel flavone aglycone content

To determine whether acid hydrolysis would increase flavone aglycone content, LC-MS/MS analyses were performed on hydrolyzed lysates of pericarps corresponding to the different maize lines (apigenin shown in Figure 3). As we determined before hydrolysis (Table 1), *P1-rr;a1* hydrolyzed lysates show the highest total flavone content at both developmental stages, when compared with all the other maize lines analyzed (~12 mg/100 gr fresh weight, Table 2). *P1-rr* also has high levels, yet ~10 fold lower than in *P1-rr;a1* (900 μg/100 gr fresh weight, Table 2). No significant differences in the total levels of hydrolyzed flavones were observed in *P1-rr;a1* at 14 and 25 DAP, whereas a 3 fold decrease was observed for *P1-rr* at 25 DAP. In contrast, both *P1-ww* and sweet corn have similarly lower levels of total flavones, which remained stable during development (9 and 17 μg/100 gr fresh weight, respectively) (Table 2).

**Table 2:**
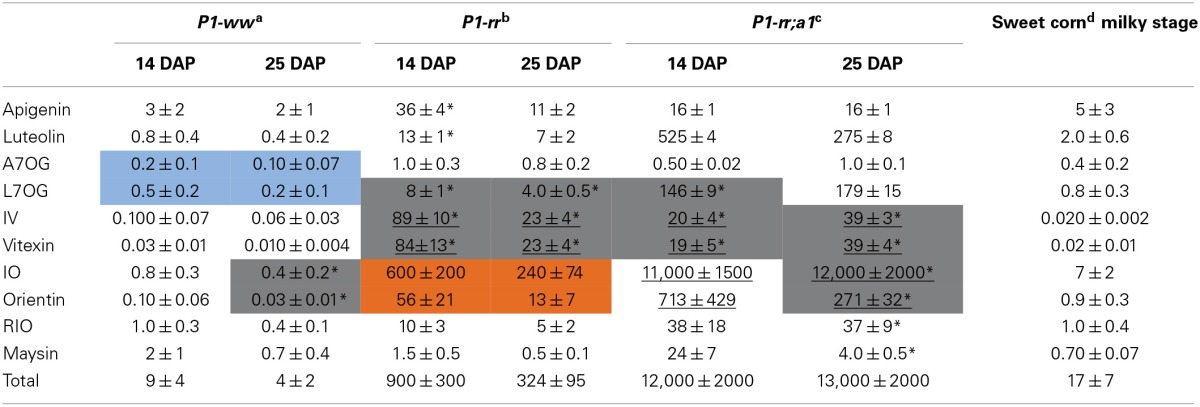
**Flavone content in hydrolyzed whole maize kernel**.

Flavones were determined by LC-MS/MS using MRM in the negative mode following parental/daughter transitions corresponding to flavones in their aglycone and glycosylated forms after acid hydrolysis (Figure 4). Results are indicated as μg of flavones/100 grams of fresh kernels. t-test values ^*^p < 0.01 compared to sweet corn. Mean values ± s.e.m.; biological replicates for a: n = 5; b: n = 5; c: n = 2 and d: n = 3. A7OG, apigenin-7-O-glucoside; L7OG, luteolin-7-O-glucoside; IV, isovitexin; IO, isoorientin and RIO, rhamnosylisoorientin.

The hydrolysis of *P1-rr* kernel extracts at 14 DAP shows a 110 fold increase in apigenin and a 65 fold increase in luteolin (36 and 13 μg/100 gr fresh weight, respectively), and these levels of flavones are about half of those observed in 25 DAP kernel hydrolyzed lysates. In *P1-rr;a1*, the aglycone luteolin is found more abundantly, reaching ~525 and 275 μg/100 gr fresh weight at 14 and 25 DAP, respectively, whereas apigenin levels are 30 times lower (16 μg/100 gr fresh weight at both developmental stages; Table 2). Hydrolysis of *P1-ww* and sweet corn extracts results in an increase of aglycones of around one order of magnitude (Table 2), likely derived from the low levels of glycosides present in these lines (Table 1).

In the case of A7OG, a different distribution is found in both *P1-rr;a1* and *P1-rr*, as compared with *P1-ww* or sweet corn. A7OG content is reduced by ~10 fold in hydrolyzed extracts obtained from 14 DAP *P1-rr* and *P1-rr a1* kernels. In contrast, increased levels are observed in both *P1-ww* and sweet corn (0.2 and 0.4 μg/100 gr fresh weight at 14 DAP; blue boxes in Table 2). If the acid hydrolysis would have been complete, A7OG and L7OG should have disappeared from the hydrolyzed samples, or their levels should have been significantly reduced, which is not always the case (Table 2). It is possible that we misidentified A7OG and L7OG. However, we do not think that this is likely because we used pure commercial standards for these two compounds, which show over a minute difference in their retention times, and they present different parental/daughter ion combinations (Supplementary Table 1). Alternatively, it is possible that the hydrolysis was not complete, despite the strong conditions used. The increase in both flavone glucosides with hydrolysis can be explained by a more decorated flavone, perhaps not present among the ones studied here (Table 1), being partially hydrolyzed into A7OG or L7OG. This is consistent with the significant increase in isoorientin after hydrolysis; none of the flavones described in Table 1 was found to be high enough in sweet corn to provide the levels of this *C*-glycosylflavone (7 μg/100 gr fresh weight) found after hydrolysis (Table 2).

L7OG decreases by ~7 and 2.5 fold at 14 and 25 DAP, respectively in *P1-rr;a1* (Tables 1, 2). However, in *P1-ww* and sweet corn, L7OG shows an increase of ~10 fold at each developmental stage (0.5 and 0.2 μg/100 gr fresh weight at 14 and 25 DAP, respectively). In *P1-rr*, L7OG levels increase ~3 fold at 14 DAP and 6 fold at 25 DAP (Table 2, gray boxes), reaching 8 and 4 μg/100 gr fresh weight, respectively. Hydrolysis increases the levels of the apigenin *C*-glucosides vitexin and isovitexin in all the samples, at both developmental stages (Table 2). *P1-rr* shows the highest levels at 14 DAP (89 and 84 μg/100 gr fresh weight) corresponding to a 10-fold increase (Table 2, gray boxes and underlined values), and the increase was even larger (~15 fold) at 25 DAP (Table 2 gray boxes and underlined values), reaching ~23 μg/100 gr fresh weight for both compounds. Vitexin and isovitexin levels also increase during hydrolysis, but to a lower level in *P1-rr;a1* at both 14 and 25 DAP (Table 2, gray boxes and underlined values), resulting in 2–4 fold differences, when compared with the non-hydrolyzed material (Table 1). In *P1-ww*, at both developmental stages as well as in sweet corn, the levels increase, but remain always significantly lower than in *P1-rr* and *P1-rr;a1*. Orientin presented the highest levels in *P1-rr;a1* at both developmental stages (underlined and in gray in Table 2), with 0.71 mg per 100 gr fresh weight at 14 DAP and 0.27 mg per 100 gr fresh weight at 14 and 25 DAP, respectively. The highest levels of isoorientin are found in *P1-rr;a1*, reaching 11 and 12 mg/100 gr fresh weight, at 14 and 25 DAP, respectively, corresponding to a ~8 fold increase. In *P1-rr*, isoorientin increases 60 fold (600 μg per 100 gr fresh weight) at 14 DAP in contrast to a 110 fold increase (240 μg per 100 gr fresh weight) at 25 DAP (Table 2, orange boxes). The orientin levels increase 6 and 8 fold at 14 and 25 DAP, respectively (56 and 13 μg/100 gr fresh weight; Table 2, orange boxes). Overall, these results suggest that additional *O*-glycosylated flavones, products of alternative metabolic pathways likely not controlled by *P1*, may be present in these lines (see Discussion).

### Maize whole-food extract preparations increased flavone aglycone content

**Figure 5:**
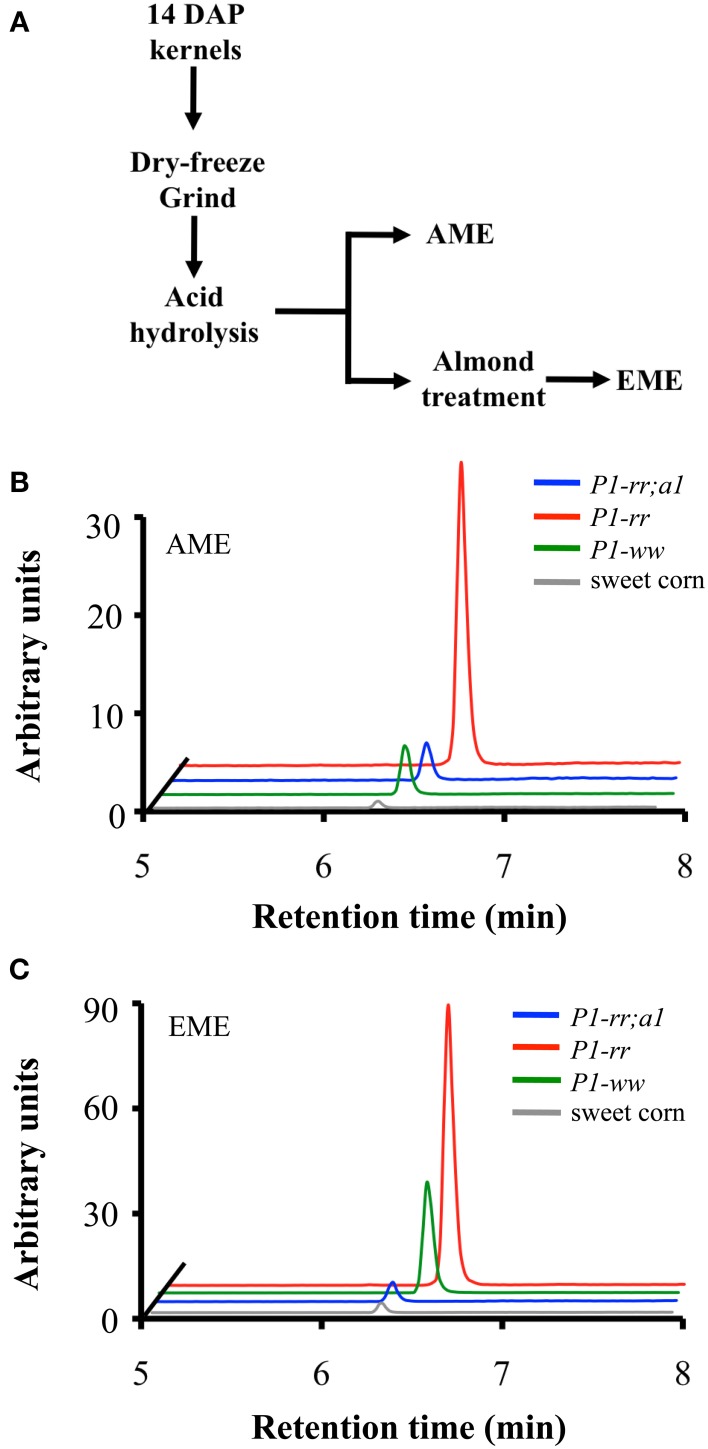
**Apigenin content in whole-food maize formulations. (A)** Work flow of whole-maize kernel food extract preparation, AME: acid whole maize extract and EME: enzymatically treated whole maize extract. Chromatograms corresponding to apigenin content from *P1-rr* (red); *P1-rr;a1* (blue); *P1-ww* (green) and sweet corn (gray) in AMEs **(B)** and EMEs **(C)**. Arbitrary units correspond to peak intensity for the 268.9/117.0 transitions in counts per second.

We previously showed that flavone aglycones have more anti-inflammatory activity than their glycosides counterparts (Hostetler et al., 2012). Hence, to evaluate whether food processing could increase the aglycone content in maize foods, we prepared whole maize extracts from kernels of the various genetic stocks using acid extraction [referred as AME (acid treated maize extracts)], or acid extraction combined with almond powder [referred as EME (enzyme treated maize extracts)], which we previously reported provides β-glucosidase activity, thereby increasing aglycone accumulation (Figure 5A) (Hostetler et al., 2012, 2013). Whole food extracts from *P1-rr* showed the highest apigenin content, reaching ~77.4 μg/100 gr fresh weight in AME and increasing ~4 fold in EME preparations to ~241.1 μg/100 gr fresh weight in EME (Figures 5B,C and Table 3). The almond powder contributes with negligible amounts of apigenin, as determined by HPLC analyses (1.4 μg/100 gr fresh weight) (Supplementary Figure 3). Both AME and EME foods from *P1-rr;a1* contain significant less apigenin, ranging from 10 to 16 μg/100 gr fresh weight, respectively (Figure 5, Table 3).

**Table 3:** **Apigenin and luteolin contents in whole-food maize extracts**.

	**AME**				**EME**			
	***P1-ww***	***P1-rr***	***P1-rr;a1***	**Sweet Corn**	***P1-rr***	***P1-ww***	***P1-rr;a1***	**Sweet corn**
Apigenin	23.9	77.4	10.0	2.0	103.9	241.1	16.0	8.2
Luteolin	15.6	184.4	279.3	5.0	18.9	300.6	274.3	7.6

Apigenin and luteolin content was determined by LC-MS/MS using MRM in the negative mode following specific parental/daughter transitions (Figures 5B,C). Results are indicated as μg of apigenin/100 grams of fresh kernels.

Surprisingly, *P1-ww* AME foods have 23.9 μg/100 gr fresh weight of apigenin, increasing 5 fold to ~103.9 μg/100 gr fresh weight in EME preparations (Figures 5B,C). In contrast, sweet corn accumulates the lowest amount of apigenin in both AME and EME (~2.0 and 8.2 μg/100 gr fresh weight for each formulation respectively, Table 3). Collectively, these results suggest that preparations of foods using maize lines with increased flavone content significantly enhance the concentration of anti-inflammatory forms of flavones lacking in commercial sweet corn.

## Discussion

In this study, we show that maize lines harboring the *P1-rr* allele in combination with recessive *a1* accumulate flavones at levels comparable to those present in celery, a vegetable often associated with the beneficial effects alleged to the Mediterranean's diet, and which is often presented as apigenin-rich (Hertog et al., 1992). Moreover, maize offers great advantages including the absence of other toxic compounds such as the linear furanocoumarins (e.g., psoralen, bergapten) that characterize celery, parsley and many of the other flavone-rich Apiaceaes and that can result in skin disorders, including cancer (Beier and Oertli, 1983; Diawara et al., 1995; Nigg et al., 1997). The ability to increase flavone content in crops of high agronomic relevance such as maize furnishes unique opportunities to improve nutrition.

At 14 DAP in *P1-rr* and *P1-rr;a1* maize kernels, about 95% of all the flavones investigated here are present in the form of *C*-glucosides, 3% as *O*-glucosides and less than 0.1% as aglycones (Table 1). A number of biological activities have been attributed to *C*-glycosylflavones (Choi et al., 2013), including anti-diabetic, potential anti-Alzheimer's disease, and anti-inflammatory activity, although aglycones are significantly more effective for the latter. Moreover, the economical value of *C*-glycosylated flavonoids is in the rise, as organic chemists are increasingly using these compounds as scaffolds for the generation of bioactive compounds because of their high biological activity potential (Talhi and Silva, 2012). However, it is not known to what extent *C*-glycosylflavones will be absorbed by the digestive system, or to what level gut microorganisms are able to process these compounds (Courts and Williamson, 2013). In contrast, *O*-glycosylflavones are readily processed by the gut microbiota and are capable of releasing the respective aglycones (Courts and Williamson, 2013). In addition, during food processing, particularly when using combinations of whole foods rich in glycosidases, an abundant release of the aglycones is observed (Figure 5). As previously noted, functional foods with increased aglycone content provide higher anti-inflammatory efficacy and increased absorption of flavonoids *in vivo* (Hostetler et al., 2012).

While the pericarp is part of the grain consumed as sweet corn, canned corn, and popcorn, it is eliminated by nixtamalization during the preparation of cornmeal for tortillas, tortilla chips and tamales. *P1-rr* kernels accumulate significantly larger flavone quantities than most lines commercially used, and the increase in free flavone and flavone *O*-glucoside accumulation in *P1-rr;a1* offers attractive opportunities to further increase maize nutritional value. The absence of the DFR enzymatic activity in *P1-rr;a1* lines diverts the naringenin intermediate toward flavone formation (Figure 1). Although a significant portion of the total flavones is used in the formation of *C*-glycosylflavones, a significant portion is utilized for the formation of flavone *O*-glucosides (Table 1), which can be hydrolyzed to the respective aglycones (Table 2). It is noteworthy, however, that hydrolysis did not completely eliminate the flavonoid *O*-glycosides (Table 2), something that was particularly puzzling in the case of *P1-ww* and the sweet corn line, in which the quantities of the flavones present fail to explain the levels observed after hydrolysis (Tables 1, 2). This likely indicates that there are a number of other flavone glycosides present in these maize lines that we failed to detect with our targeted MRM approach that could be identified through untargeted metabolomics analyses. A large number of flavones (more than 28, including apigenin, apigenin-8-*C*-glucoside, vitexin and A7OG) were recently identified in a genome-wide association study of kernels from a large number of maize inbred lines using untargeted metabolomics (Wen et al., 2014). Perhaps one of these other flavones is responsible for the formation of small quantities of A7OG, L7OG, and isoorientin after hydrolysis. Additional studies will be required to determine if this is the case. There are multiple ways in which consumers could benefit from immature (14–20 DAP) high-flavone corn kernels, once the appropriate stocks are in the market, for example as popcorn, sweet corn, and canned corn. However, our studies also start providing some possible paths forward to introduce combinations of mutations that might significantly increase the accumulation of flavone in mature, dry kernels. *Salmon silk* (*Sm*) genes (Anderson, 1921) are required for the formation of the major *C*-glycosylflavones present in maize floral organs (McMullen et al., 2004). Presumably, the combination of *a1* and *sm* mutations in a *P1-rr* background will provide a further opportunity to increase the accumulation of hydrolyzable flavones in the maize kernel.

As previously shown in rice and wheat (Brazier-Hicks et al., 2009), and more recently in maize (Morohashi et al., 2012), the formation of *C*-glycosylflavones involves hydroxylation of a flavanone to a 2-hydroxyflavanone by F2H, which can then be *C*-glycosylated (Brazier-Hicks et al., 2009; Falcone Ferreyra et al., 2013). This poses the question of how apigenin or luteolin are formed, which presumably serve as the substrates for the respective *O*-glycosides. We speculate that there is a *bona fide* maize flavone synthase that can use naringenin (or eriodctyol) as substrates to form apigenin (or luteolin). Indeed, such an enzyme might not be significantly controlled by P1, since the levels of apigenin in *P1-ww* and *P1-rr* are comparable (Table 1). To determine to what extent the genes encoding the enzymes necessary for the formation of the flavanones are expressed in *P1-ww*, we analyzed recent RNA-Seq results obtained from pericarps with contrasting *P1* alleles (Morohashi et al., 2012). We found that, albeit at low levels, two chalcone synthase genes (encoded by *C2* and *WHP*) and two chalcone isomerase genes (Supplementary Figure 4) are expressed in *P1-ww* pericarps at 14 and 25 DAP. These results suggest that a *P1*-independent flavone accumulation pathway might be active in maize floral tissues.

In summary, our studies demonstrate that *P1-rr* maize kernels at 14 DAP potentially provide a good source of flavones that could complement flavone-deficient diets in the absence of other flavone-rich vegetables. The rich diversity offered by maize inbred lines and cultivars provide unique opportunities to select for maize genetic stocks that are simultaneously more resistant to emerging pathogens associated with global climate changes, and with increased levels of flavones with alleged beneficial effects and improved nutritional qualities.

## Author contributions

Erich Grotewold, María I. Casas and Andrea I. Doseff carried out the experimental design. María I. Casas and Silvia Duarte performed the experiments. All authors analyzed data and contributed in the writing of the manuscript.

### Conflict of interest statement

The authors declare that the research was conducted in the absence of any commercial or financial relationships that could be construed as a potential conflict of interest.

## Abbreviations

A7OG: apigenin-7-*O*-glucoside
L7OG: luteolin-7-*O*-glucoside
IV: isovitexin
IO: isoorientin
RIO: rhamnosylisoorientin
HPLC: high performance liquid chromatography
LC-MS/MS: liquid chromatography followed by tandem mass spectrometry
AME: acid whole maize extract
EME: enzymatically treated whole maize extract.
