# Supplementary material for: Flavone-rich maize: an opportunity to improve the nutritional value of an important commodity crop
Source: Front Plant Sci. 2014 Sep 8;5:440. doi: 10.3389/fpls.2014.00440 (PMC4157551; doi:10.3389/fpls.2014.00440)
Supplement: Supplementary file 1 [file Presentation1.PDF]

Analyte	Parent ion (amu)	Daughter ion (amu)	Rt (min)	DP (V)	EP (V)	CE (V)	CXP (V)
Apigenin	268.9	117.0	6.4	-145.0	-10.0	-34.0	-11.0
Luteolin	284.9	151.0	5.5	-170.0	-10.0	-36.0	-41.0
A7OG	430.9	269.0	3.8	-170.0	-10.0	-40.0	-29.0
L7OG	447.1	285.1	2.6	-200.0	-10.0	-46.0	-31.0
IV	430.9	283.0	2.2	-250.0	-10.0	-46.0	-31.0
IO	447.0	357.0	1.7	-170.0	-10.0	-30.0	-23.0
V	430.9	311.0	2.2	-170.0	-10.0	-32.0	-37.0
IV	447.0	327.0	1.7	-165.0	-10.0	-38.0	-29.0
RIO	593.1	298.0	1.3	-150.0	-10.0	-60.0	-1.0
Maysin	575.0	411.1	4.2	-115.0	-10.0	-30.0	-37.0

Supplementary table 1. Summary of LC-MS/MS conditions of the compounds presented in this study. Parental ion (Q1) and daughter ion (Q3) transitions; retention times (Rt); declustering potential (DP); entrance potential (EP); collision energy (CE) and collision exit potential (CPX) for each compound used in this study. A7OG: apigenin-7-*O*-glucoside; L7OG: luteolin-7-*O*-glucoside; IV: isovitexin; V: vitexin; IO: isoorientin ; O: orientin and RIO: rhamnosylisoorientin.

	Apigenin	Luteolin	A7OG	L7OG	IV	V	IO	O	RIO	Maysin
**LQ (fmol)**	49.30	10.90	3.50	13.20	2.10	0.10	29.80	4.90	3.70	13.20
**LD (fmol)**	14.80	3.30	1.10	3.90	0.60	0.03	8.90	1.50	1.10	3.90
**R^2**	1.000	0.996	0.996	0.995	0.995	0.998	0.992	0.986	0.992	0.999
**Exact mass (amu)**	270.05	286.05	432.11	448.10	432.11	432.11	448.10	448.10	594.16	576.15

Supplementary table 2. Summary of LC-MS/MS standard curves of the flavones used in this study. Limit of quantification (LQ), limit of detection (LD) and R2 from each standard curve. A7OG: apigenin-7-*O*-glucoside; L7OG: luteolin-7-*O*-glucoside; IV: isovitexin; V: vitexin; IO: isoorientin ; O: orientin and RIO: rhamnosylisoorientin.

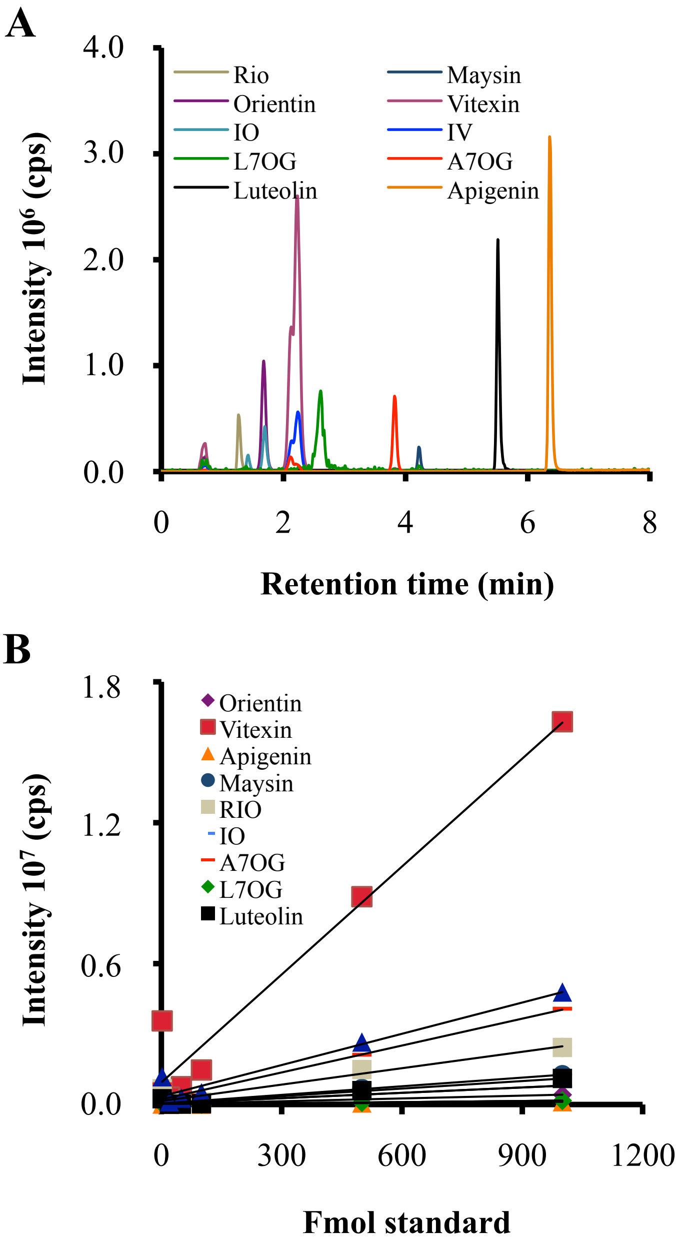

Supplementary figure 1. LC-MS/MS chromatograms of flavones standards and corresponding standard curves. (A) LC-MS/MS chromatograms of apigenin; luteolin; vitexin; orientin; isoorientin (IO); isovitexin (IV) ; apigenin-7-*O*-glucoside (A7OG); luteolin-7-*O*-glucoside (L7OG); maysin and rhamnosylisoorientin (RIO). (B) LC-MS/MS standard curves for LC-MS/MS for flavones used in (A).

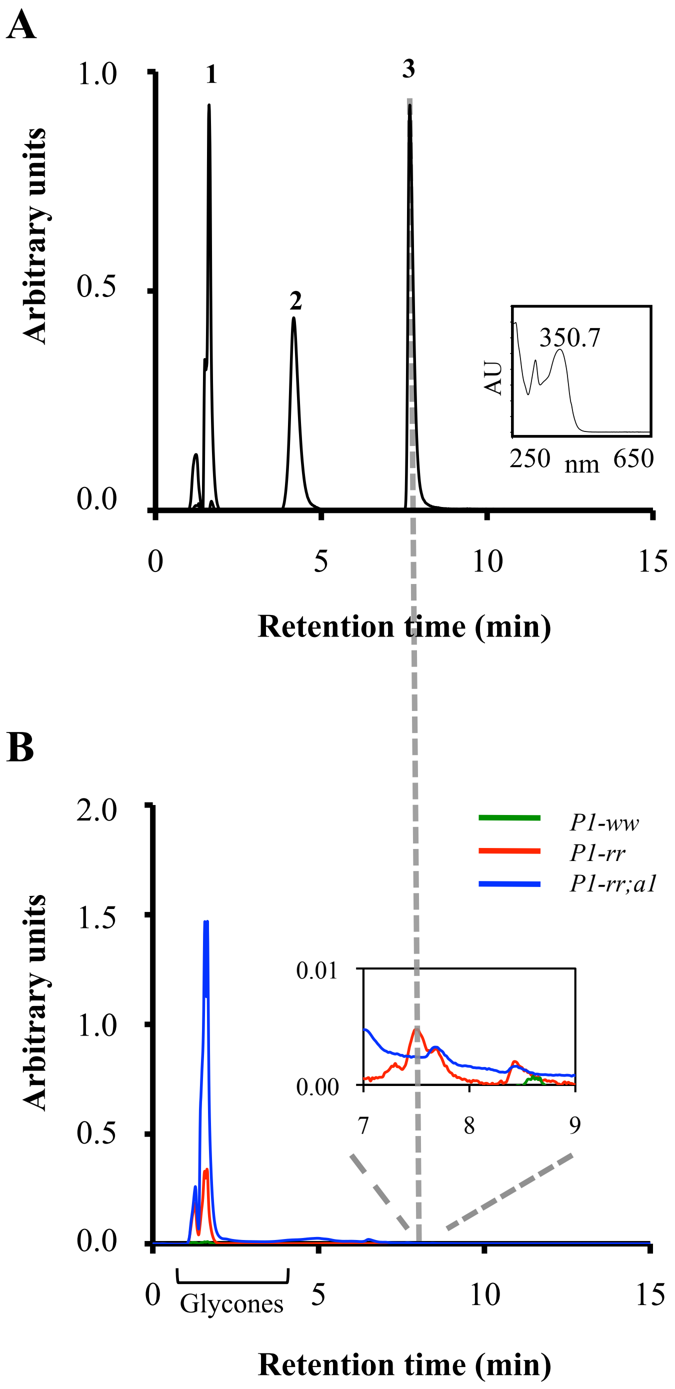

Supplementary figure 2. HPLC analyses of flavones at 14 DAP. HPLC chromatograms were obtained at 350 nm. (A) Isovitexin (1); apigenin-7-O-glucoside (2) and apigenin (3) standards. Inset shows the absorption spectrum of these compounds. (B) Chromatograms of *P1-ww* (green), *P1-rr* (red), and *P1-rr;a1* (blue) kernels at 14 DAP. Inset shows the content of apigenin in the lines studied.

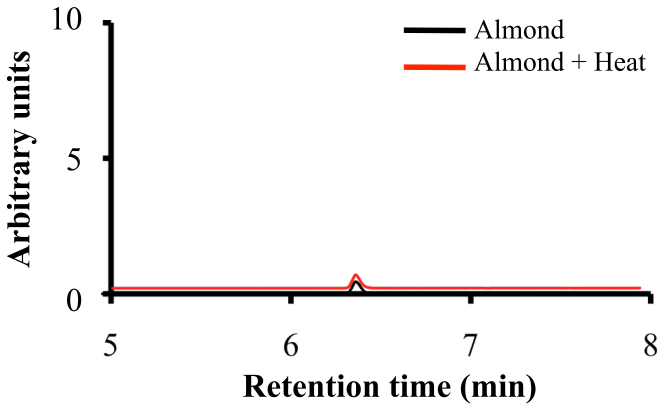

Supplementary figure 3. Apigenin content in raw almond powder processed following the steps of preparation of maize whole food extracts with and without heat treatment. The chromatograms depict apigenin content in raw almond powder (black) and raw almond powder incubated at 50°C (red) following the same steps used to prepare the whole-maize food extracts.

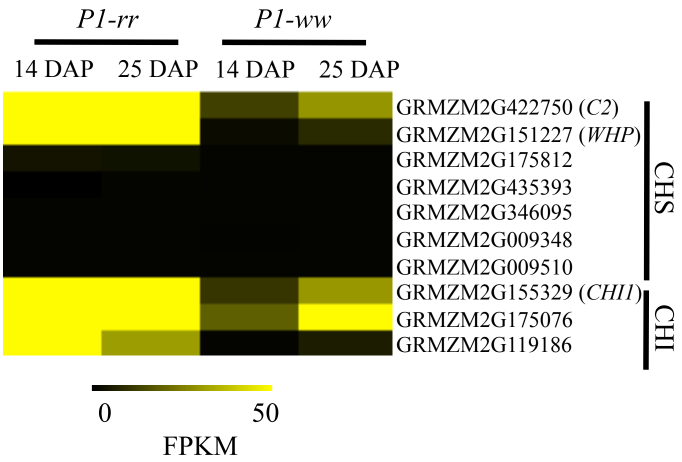

Supplementary figure 4. Expression of genes corresponding to the initial steps in the flavonoid biosynthetic pathway. Heatmap indicating the expression of chalcone synthase (CHS) and chalcone isomerase (CHI) genes in pericarps of *P1-rr* and *P1-ww* pericarps at two developmental stages as determined by high throughput transcriptome analysis (RNA-Seq). Transcript levels for each gene are expressed in number of fragments per kilo-base of transcript sequence per million base pairs sequenced (FPKM). GRMZM gene identifiers as used in Gramene (www.gramene.org). Data adapted from Morohashi et al, 2012.
